# Cardiac Masses on Cardiac CT: A Review

**DOI:** 10.1007/s12410-014-9281-1

**Published:** 2014-06-17

**Authors:** David Kassop, Michael S. Donovan, Michael K. Cheezum, Binh T. Nguyen, Neil B. Gambill, Ron Blankstein, Todd C. Villines

**Affiliations:** 1 Cardiology Service, Walter Reed National Military Medical Center, 8901 Wisconsin Avenue, Bethesda, MD 20889 USA; 2 Department of Radiology, Walter Reed National Military Medical Center, 8901 Wisconsin Avenue, Bethesda, MD 20889 USA; 3 Departments of Medicine and Radiology (Cardiovascular Division), Brigham and Women’s Hospital, Non-Invasive Cardiovascular Imaging Program, Boston, MA 02115 USA

**Keywords:** Cardiac computed tomography, Coronary computed tomographic angiography, Cardiac mass, Neoplasm, Tumor, Myxoma, Lipoma, Teratoma, Sarcoma, Metastasis, Thrombus, Pericardial cyst

## Abstract

Cardiac masses are rare entities that can be broadly categorized as either neoplastic or non-neoplastic. Neoplastic masses include benign and malignant tumors. In the heart, metastatic tumors are more common than primary malignant tumors. Whether incidentally found or diagnosed as a result of patients’ symptoms, cardiac masses can be identified and further characterized by a range of cardiovascular imaging options. While echocardiography remains the first-line imaging modality, cardiac computed tomography (cardiac CT) has become an increasingly utilized modality for the assessment of cardiac masses, especially when other imaging modalities are non-diagnostic or contraindicated. With high isotropic spatial and temporal resolution, fast acquisition times, and multiplanar image reconstruction capabilities, cardiac CT offers an alternative to cardiovascular magnetic resonance imaging in many patients. Additionally, cardiac masses may be incidentally discovered during cardiac CT for other reasons, requiring imagers to understand the unique features of a diverse range of cardiac masses. Herein, we define the characteristic imaging features of commonly encountered and selected cardiac masses and define the role of cardiac CT among noninvasive imaging options.

## Introduction

Cardiac masses are uncommon findings and can be categorized as either neoplastic or non-neoplastic (Table 1). Neoplastic cardiac masses are comprised of primary benign tumors, primary malignant tumors, or metastatic tumors. Primary cardiac tumors are exceedingly rare with a reported prevalence of 0.001 to 0.03 % in an autopsy series, while metastatic tumors to the heart are reported to be 20 to 40 times more common [1]. Of all cardiac tumors that originate in the heart (primary), approximately 75 % are benign, with myxoma accounting for at least half of reported cases [1, 2]. The remaining 25 % of primary cardiac tumors are malignant with sarcoma accounting for a majority of reported cases [3]. Non-neoplastic cardiac masses, such as thrombi, pericardial cysts, and prominent anatomic structures, can often mimic cardiac tumors. The evaluation of cardiac masses may therefore be a diagnostic challenge.

**Table 1:** Cardiac neoplasms and commonly-encountered non-neoplastic masses

Cardiac masses	Type
**Benign tumors**	Myxoma
	Lipoma
	Papillary fibroelastoma
	Fibroma
	Hemangioma
	Paraganglioma
	Teratoma
	Atrioventricular (AV) nodal mesothelioma
	Rhabdomyoma
**Malignant tumors**	Metastatic
	Angiosarcoma
	Rhabdomyosarcoma
	Fibrosarcoma
	Lymphosarcoma
	Osteosarcoma
	Liposarcoma
	Mesothelioma
**Non-neoplastic**	Lipomatous hypertrophy of interatrial septum
	Intracardiac thrombus
	Pericardial cyst
	Large coronary artery aneurysm
	Valvular vegetation
	Crista terminalis

## Imaging Approach to Cardiac Masses

Noninvasive imaging plays a critical role in the diagnosis and surgical planning of cardiac masses. Furthermore, certain characteristics identified on imaging may help distinguish neoplastic versus non-neoplastic masses and benign versus malignant tumors (Table 2). Despite the versatility and high accuracy of cardiac computed tomography (cardiac CT) and cardiac magnetic resonance (MR) imaging, echocardiography remains the first-line for cardiac mass evaluation because of its widespread availability, lack of iodinated contrast material or radiation exposure, and its dynamic assessment of cardiac masses in relation to the surrounding chambers, valves, and pericardium. However, echocardiography provides limited assessment of soft-tissue characteristics and extracardiac structures and may be limited by poor acoustic windows, particularly in obese patients and those with chronic lung disease [4•].

**Table 2:** Cardiac CT features of benign and malignant tumors

Feature	Benign	Malignant
Size/number	Small (<5 cm), single lesion	Large (>5 cm), multiple lesions
Location	Left > > right	Right > > left
Morphology	Intracameral	Intramural
Attachment	Narrow stalk, pedunculated	Broad base
Enhancement	Absent to minimal	Modest to intense
Margin	Smooth, well-defined	Irregular, ill-defined
Invasion	None	Intra-/extracardiac infiltration
Metastasis	None	May be present
Pericardial effusion	None	May be present
Calcification	Rare (except for small foci in fibroma, myxoma, or teratoma)	Large foci in osteosarcoma

Cardiac CT and MR are often utilized synergistically with echocardiography in the evaluation and management of cardiac masses. Appropriate Use Criteria for cardiac MR [5] and cardiac CT [6] and an Expert Consensus statement for pericardial disease imaging [7••] provide specific guidance on the approach to cardiac masses (Table 3). Cardiac MR is often the preferred imaging modality for cardiac masses because of its superior soft-tissue characterization, high temporal resolution, multiplanar imaging capabilities, and unrestricted field of view [8]. Since MR does not require the use of ionizing radiation, it is the modality of choice, along with echocardiography, for pediatric patients with cardiac masses. However, cardiac MR is dependent on patient cooperation to obtain quality images and is specifically contraindicated in patients with claustrophobia and implanted magnetic devices. At times, MR may also be limited for evaluating small mobile masses (e.g., papillary fibroelastoma or valvular vegetations) due to limitations in spatial resolution and typically does not provide detailed assessment of the coronary arteries in cases where the assessment of coronary artery disease prior to surgery is an important clinical question.

**Table 3:** Recommendations for the use of noninvasive imaging to evaluate suspected cardiac and pericardial masses

**2006 Appropriate use criteria for cardiac MRI** [5] - Evaluation of cardiac mass (suspected tumor or thrombus) ……… **Appropriate** - Evaluation of pericardial mass ……………………………………. **Appropriate**
**2010 Appropriate use criteria for cardiac CT** [6] - *Initial* evaluation of cardiac mass (suspected tumor or thrombus) … **Inappropriate** - Evaluation of cardiac mass (suspected tumor or thrombus) [when] inadequate images from other noninvasive methods …….… **Appropriate** - Evaluation of pericardial anatomy………………………………….. **Appropriate**
**2013 Expert consensus for pericardial disease imaging** [7••] - “Echocardiography is the initial imaging test to assess pericardial masses” - “CT and/or MRI should be done for better tissue characterization of the mass and detection of metastasis (if malignancy is suspected).”

CT, computed tomography; MRI, magnetic resonance imaging

Cardiac CT is an alternative imaging modality to assess cardiac masses, particularly in patients with known contraindications to MR or in patients with inadequate images from other noninvasive methods. Cardiac CT is a fast imaging technique with electrocardiographic (ECG) gating that provides high quality images with superior spatial resolution. Electrocardiographic gating minimizes motion-related artifacts and allows a more precise delineation of the lesion margins. Compared to other cardiac imaging modalities, CT is optimal for the evaluation of calcified masses, the global assessment of the chest and lung tissue and corresponding vascular structures, and the exclusion of obstructive coronary artery disease or masses which involve the coronary arteries. Significant disadvantages with CT include radiation exposure, a small risk of contrast-induced nephropathy, and lower soft tissue and temporal resolutions as compared with magnetic resonance imaging.

Cardiac CT is also useful to detect metastases in suspected malignancies especially when coupled with ^18^ F-fluorodeoxyglucose (FDG) positron emission tomography (PET). The ability of ^18^ F-FDG PET/CT to detect the increased metabolism of glucose may help distinguish malignancy from a benign neoplasm. For example, primary malignant cardiac tumors and metastatic tumors show significantly higher glucose uptake as quantified by ^18^ F-FDG PET/CT standardized uptake value (SUV) than primary benign cardiac tumors. This differentiation may improve the detection of distant metastases, especially when the results will be used to impact therapy [9•].

The formulation of a differential diagnosis for cardiac masses on cardiac CT is based on several key aspects. They include size, quantity, location (cardiac chamber, pericardial involvement, extracardiac structures), morphology (attachment, margin appearance, infiltration), tissue characteristics (calcification, fat attenuation, vascularity), and clinical correlation (known malignancy or infection, presence of a catheter, associated syndromes) (Table 4). When performed to assess for cardiac masses, specific CT protocols unique from those typically utilized for coronary imaging can be implemented to obtain high quality images. For example, right atrial or right ventricle masses may require a right heart contrast injection protocol with multiphasic contrast administration to obtain optimal images. Additionally, low dose non-contrast and delayed CT imaging may be helpful in distinguishing intracardiac thrombus from tumor and to detect calcifications. Clinical and imaging features of primary cardiac masses are summarized in Table 2.

**Table 4:** Cardiac CT features of primary cardiac tumors

Tumor	Location	Cardiac CT findings
**Benign (75 %)**		
Myxoma	LA > RA, ventricles	Pedunculated, mobile, heterogeneous, low attenuation, 10 % calcified; may prolapse through the mitral valve
Lipoma	Varies	Smooth, encapsulated, fat attenuation, no enhancement; multiple lesions may be seen with tuberous sclerosis
Fibroelastoma	Valves	Small (10 mm), smooth, pedunculated, mobile
Rhabdomyoma	LV > RV	Smooth, multiple, attenuation similar to myocardium; > 90 % in infants and children
Fibroma	LV > RV	Homogenous, low attenuation, minimal enhancement, central calcification; second most common in infants and children
Hemangioma	LV > RV	Heterogeneous, intense enhancement
Teratoma	Pericardium	Multicystic, moderate enhancement, partially calcified
**Malignant (25 %)**		
Angiosarcoma	RA > RV, pericardium	Broad base, irregular, heterogeneous, low attenuation, infiltrative, pericardial effusion, metastatic
Rhabdomyosarcoma	Myocardium, valves	Irregular, low attenuation, infiltrative; most common in infants and children
Fibrosarcoma	LA, pericardium	Large, irregular, low attenuation, central necrosis, infiltrative
Osteosarcoma	LA > RA, RV	Broad base, low attenuation, infiltrative, extensive calcification
Liposarcoma	LA > RA, pericardium	Large, fat and soft tissue attenuation, mild contrast enhancement, infiltrative
Mesothelioma	Pericardium	Infiltrative, variable attenuation, pericardial effusion

LA, left atrium; LV, left ventricle; RA, right atrium; RV, right ventricle

## Benign Cardiac Neoplasms

### Myxoma

Myxoma is the most common primary cardiac tumor, accounting for 25-50 % of cases. It typically affects middle-aged adults, 30-60 years of age, with a higher prevalence in women [10]. A majority (60-75 %) of cases are located in the left atrium attached to the fossa ovalis by a thin stalk, though less common locations include the right atrium (15-20 %), inferior vena cava, ventricles, and the valve leaflets [11]. Myxomas are solitary tumors that have a low risk of recurrence following surgical resection. However, multiple myxomas and myxomas with an atypical location have a higher risk of recurrence. These are more common in younger men and in several familial syndromes accounting for 10 % of cases [12, 13]:

- *Carney complex*: multiple recurrent cardiac and mucocutaneous myxomas, pigmented skin lesions, schwannomas, and endocrine overactivity neoplasms.
- *LAMB*: Lentigines, Atrial Myxoma, and Blue nevi.
- *NAME*: Nevi, Atrial myxoma, Myxoid neurofibroma, and Ephelides.

**Fig. 1:**
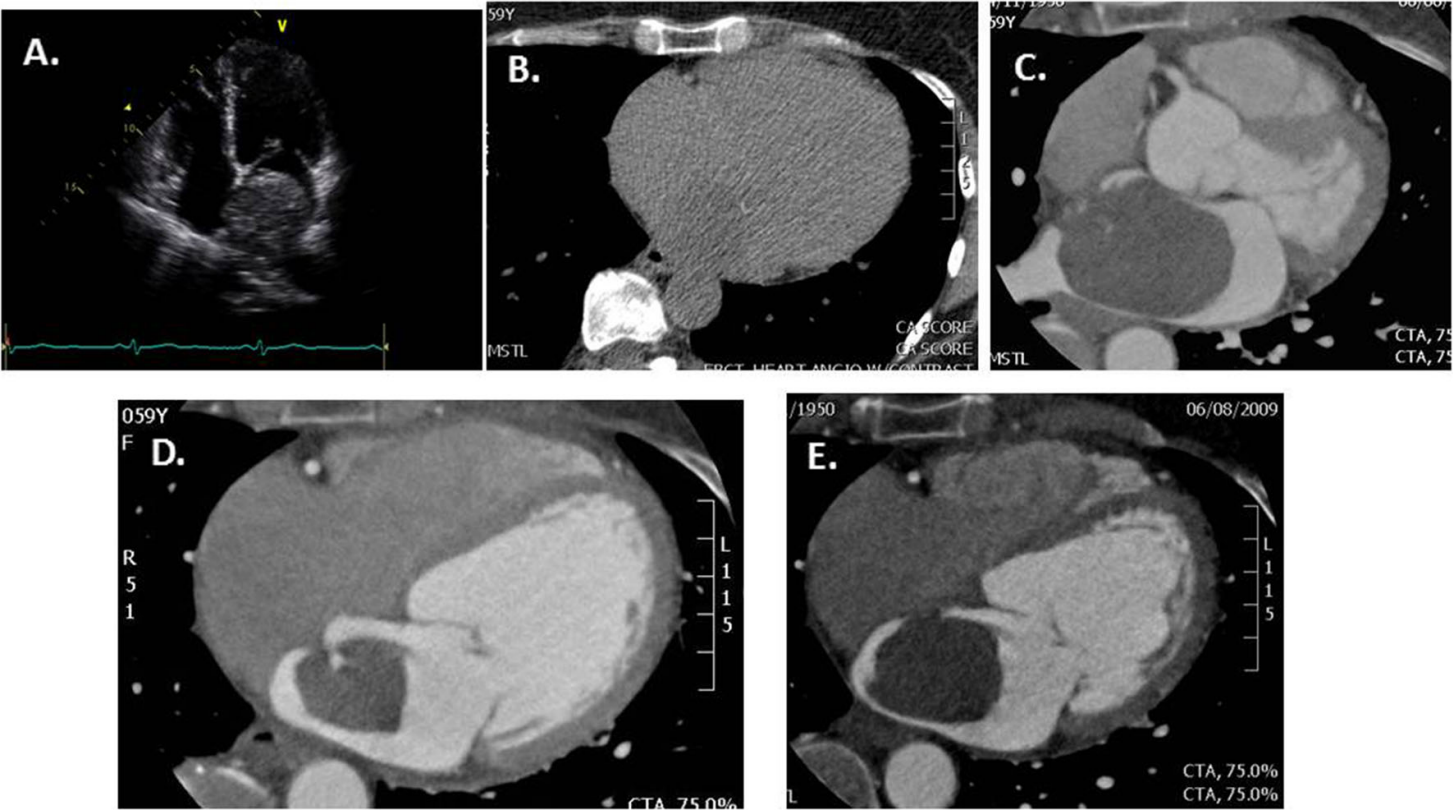
Atrial myxoma. (**a**) Apical four-chamber view on transthoracic echocardiogram demonstrating a large mass occupying the majority of the left atrial cavity. (**b**) Non-contrast axial image faintly demonstrating the large left atrial mass. (**c**-**e**) Cardiac CT angiography images demonstrating a large, low attenuating mass with a small stalk attached to the fossa ovale, consistent with a large left atrial myxoma. Note the patchy, non-homogenous contrast enhancement

On cardiac CT, approximately two-thirds of myxomas are ovoid with a smooth or lobular shape, with the remainder villous in appearance. When visualized on non-contrast CT, they typically appear hypodense, consistent with blood, and may demonstrate calcifications more often in the right atrial location (Fig. 1). On contrast-enhanced cardiac CT, myxomas appear as intracavitary filling defects with heterogeneous contrast enhancement, though the intensity may be variable depending on their chronicity and whether necrosis or hemorrhage is present. Often mobile on cine ECG gated imaging, myxomas may prolapse across the atrioventricular valves causing outflow tract obstruction depending on their size and location. When symptoms or peripheral emboli occur, surgical removal is often required.

### Lipoma

**Fig. 2:**
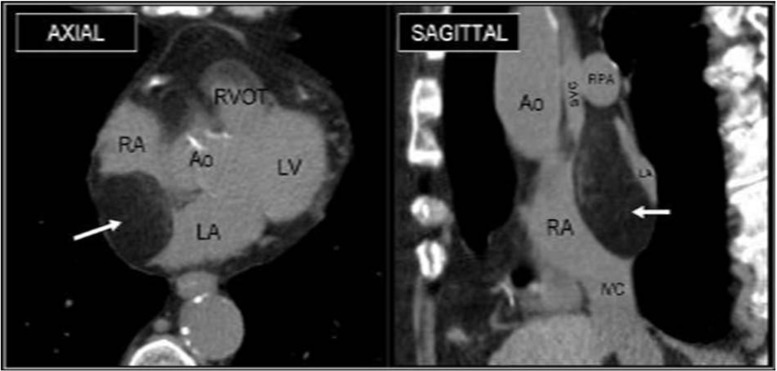
Lipoma. Non-gated MDCT images in the axial and sagittal oblique planes from a 65-year-old female incidentally demonstrate a fatty mass in the posterior and superior region of the interatrial septum consistent with an interatrial lipoma (arrow). *Right atrium (RA), Right ventricular outflow tract (RVOT), Left atrium (LA), Left ventricle (LV), Aorta (Ao) Superior vena cava (SVC), Right pulmonary artery (RPA)*. *Reproduced with permission from [17]

Lipoma is the second most common primary benign tumor and accounts for approximately 10 % of primary cardiac tumors [8]. Lipomas commonly occur in middle-aged and older adults. Lipomas are encapsulated, well-circumscribed tumors consisting of mature adipocytes that can occur anywhere in the heart (Fig. 2). Approximately 50 % arise in the epicardial or mid-myocardial layers, while the other half are subendocardial, where they create filling defects with a homogenous appearance of fat attenuation (density < -50 Hounsfield units [HU]). They can occasionally be visible as thin septations on cardiac CT. Although most are solitary tumors, multiple cardiac lipomas can occur and have been described in patients with tuberous sclerosis [10]. Classically, lipomas are benign and slow growing, but depending on their location, lipomas may rarely cause a variety of complications to include compression of the coronary arteries or pericardial space (subepicardial), outflow obstruction (subendocardial), or arrhythmias (intra-myocardial).

### Papillary Fibroelastoma

**Fig. 3:**
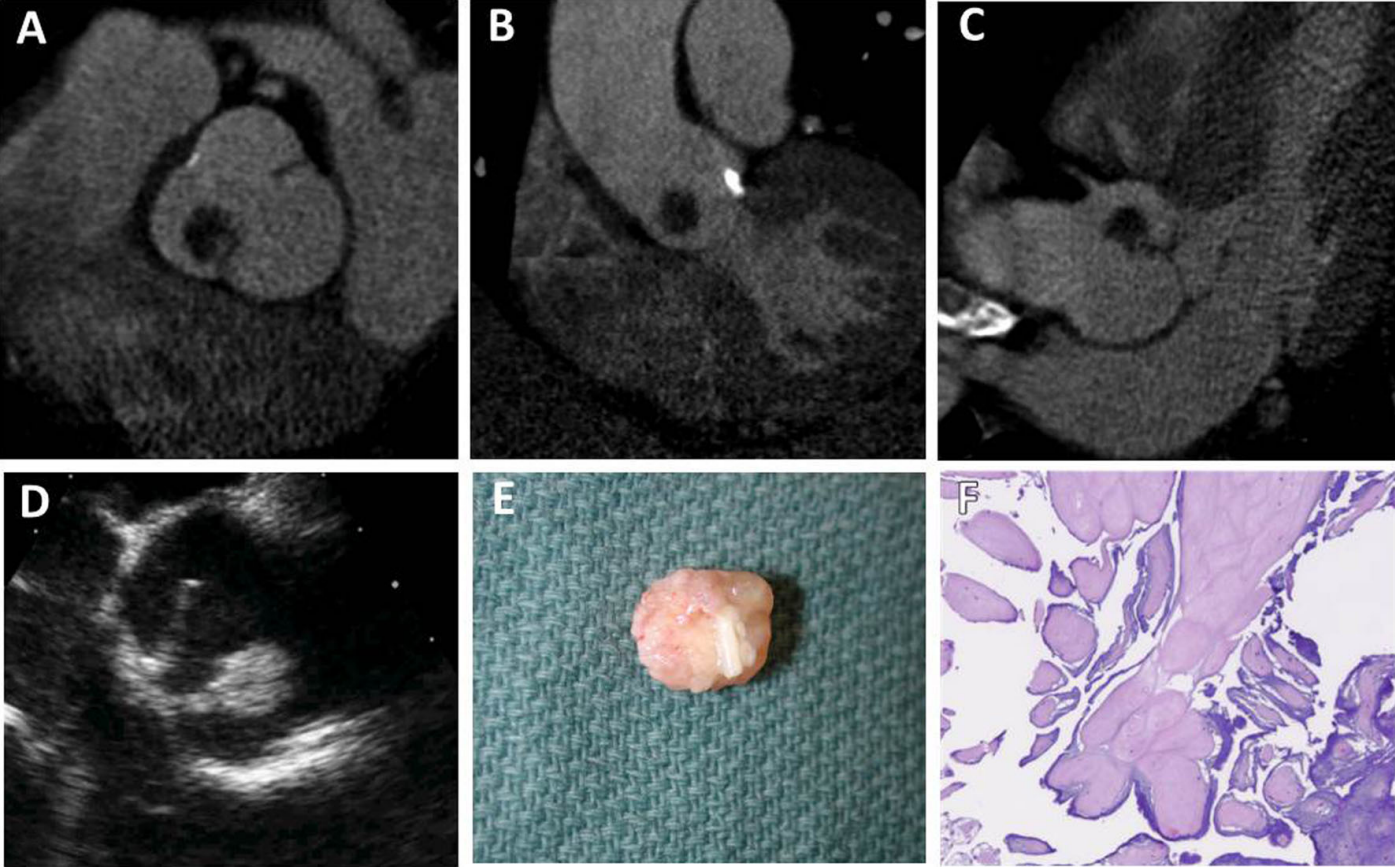
Papillary fibroelastoma. CCT demonstrates a hypodense, microlobulated mass attached via a short stalk to the aortic valve on the aortic surface of the right coronary cusp (**a**-**c**). A transesophageal echocardiogram (TEE), mid-esophageal short-axis view of the aortic root, demonstrates a mobile echodense mass attached to the right coronary cusp (**d**). Gross examination following resection revealed a 1.1 × 1.3 × 1.2 cm tan-pink, friable, soft mass with a short stalk and a micronodular appearance, a typical feature for fibroelastoma (**e**). Histopathology of the mass demonstrates an avascular core surrounded by a loose matrix with multiple adjacent fronds covered by endothelium (**f**)

Papillary fibroelastoma is the third most common primary benign cardiac tumor with an incidence of up to 0.33 % in autopsy series [14]. Papillary fibroelastomas account for approximately 75 % of all cardiac valvular tumors and affect men and women equally with a mean age of 60 years [15]. They are characterized by a collection of avascular fronds of dense connective tissue lined by endothelium and may arise from any endocardial surface, though the majority are found on the aortic and mitral valves. Most papillary fibroelastomas are solitary and small with an average diameter of 10 mm. However, some are mobile with a stalk and appear more likely to give rise to embolism [14]. Though most patients are asymptomatic, patients may experience cerebral embolic symptoms, such as transient ischemic attacks or strokes, or angina from coronary ostial obstruction from aortic valve lesions. Echocardiography is the preferred means for evaluation. Papillary fibroelastomas may be difficult to identify on cardiac CT due to their small size, pronounced mobility, and lack of calcification. If visible in cardiac CT, papillary fibroelastomas appear hypodense with irregular borders attached by a thin stalk (Fig. 3) and mobile on cine ECG gated imaging.

### Teratoma

**Fig. 4:**
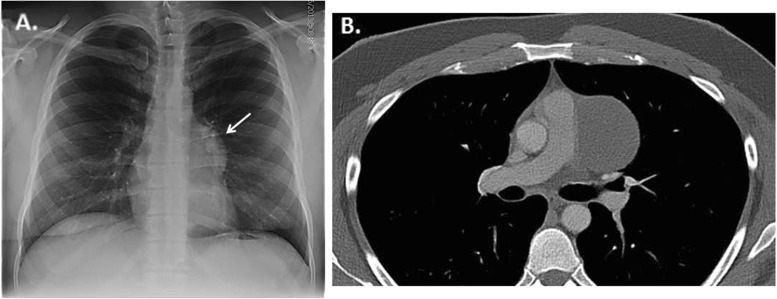
Teratoma. **a**. Posteroanterior chest radiograph demonstrating a mass in the aortopulmonary window (arrow). **b**. Contrast non-gated axial CT image demonstrating a cystic pericardial mass. Subsequent cardiac MR suggested possible solid components atypical for a simple pericardial cysts. Pathological analysis post-resection identified the mass as a teratoma

Cardiac teratoma is a rare, primary benign germ cell tumor that typically affects infants and children. Over 90 % of cardiac teratomas involve the pericardium with a predilection for pericardial effusions resulting in tamponade and/or compression of right-sided vascular structures (superior vena cava, right atrium, and pulmonary artery) [1, 2]. Cardiac teratomas contain derivatives of all three germ layers, with mature endodermal, mesodermal, and neuroectodermal elements [2]. On CT, they appear as multicystic, heterogeneous tumors typically with an associated pericardial effusion (Fig. 4) [16]. Lipid or calcific densities can also be present on CT which often provide useful clues to the diagnosis. Cardiac CT may demonstrate their intrapericardial location, extent, and relation to vascular structures to which they are adherent, assisting planned surgical interventions [16].

## Malignant Cardiac Neoplasms

### Metastatic Tumors

Malignant cardiac tumors are most often a result of metastatic disease arising by direct extension of adjacent organs or spread via hematogenous, lymphatic, or intracavitary routes [1]. The most common malignancies metastatic to the heart originate from the lung (35-40 %), followed by breast (10 %) and hematologic (10-20 %) carcinomas. Melanoma has the greatest propensity to metastasize to the heart, but it is often found late in the disease process [18•]. Other primary sites of origin include renal, hepatic, adrenal, and thyroid. The most frequent location of metastasis is the pericardium (65-70 %), followed by epicardium (25-35 %) and myocardium (30 %). Endocardial or intracavitary involvement is rarely observed (3.5 % of cases) [18•].

Cardiac CT offers several advantages for the evaluation of metastatic cardiac involvement. Primarily, cardiac CT offers the ability to image direct tumor extension and extracardiac involvement with both three-dimensional reconstruction capability and superior spatial resolution. On cardiac CT, pericardial metastasis can appear as pericardial thickening, disruption, or effusion. Myocardial involvement typically demonstrates thickening and nodularity. Solid tumors often demonstrate enhancement following the administration of intravenous contrast.

### Angiosarcoma

Angiosarcoma is the most common primary cardiac malignant tumor and is comprised of cells that develop multiple, irregular vascular channels. The primary site of origin is the right atrial free wall in 80 % of cases and less commonly the right ventricle or pericardium [19]. The tumor morphology typically consists of a large, multilobar mass with a heterogeneous composition that spreads along the epicardial surface and replaces the right atrial wall. Given the bulky nature, the tumor may comprise the majority of the right atrium and involve the right coronary artery leading to rupture [20]. These tumors may also be localized to the pericardium and often invade adjacent cardiac structures leading to cardiomegaly and recurrent pericardial effusions [21].

**Fig. 5:**
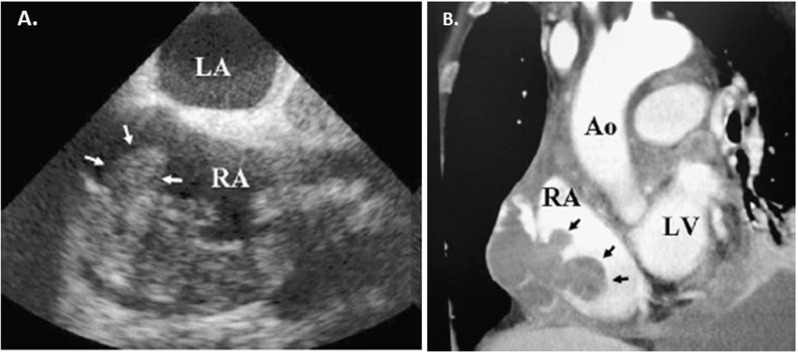
Angiosarcoma. **a**. Transesophageal echocardiogram demonstrating a large, lobulated mass in the right atrium. **b**. Contrast non-gated cardiac CT demonstrating a hypodense, multilobular mass in the right atrium. The mass invaded into the pericardial space. Aorta (Ao), Left atrium (LA), Left ventricle (LV), Right atrium (RA)

Cardiac CT allows evaluation of the tumor burden and vascularity to help distinguish this rare malignant tumor (Fig. 5). The tumor typically is characterized by a broad-based attachment with a hematogeneous connection between the right atrium and the tumor cavity which may often be identified on early imaging. Delayed imaging allows for better visualization of the tumor cavity given late contrast enhancement. The tumor is grossly hemorrhagic and often heterogeneous in appearance given the scattered areas of non-enhancing necrosis. Additionally, invasion to adjacent structures including the myocardium, pericardium, mediastinum, great vessels, and pulmonary metastasis may be identified. With pericardial involvement, there is usually “sheet-like” thickening due to the distribution and arrangement of tumor cells, in contrast to the nodular appearance seen with rhabdomyosarcomas. Pericardial and pulmonary effusions are also readily observed.

### Rhabdomyosarcoma

Rhabdomyosarcoma is the second most common primary malignant tumor. It accounts for 4-7 % of cardiac sarcomas and remains the most common pediatric cardiac malignancy [1]. It is a malignant tumor of striated muscle that always involves the myocardium. In contrast to angiosarcomas, rhabdomyosarcomas may arise from any location with no predilection for a specific cavity as 60 % of cases involve multiple sites of origin [22]. Additionally, the tumors may invade the pericardial space with a characteristic nodular appearance [23].

**Fig. 6:**
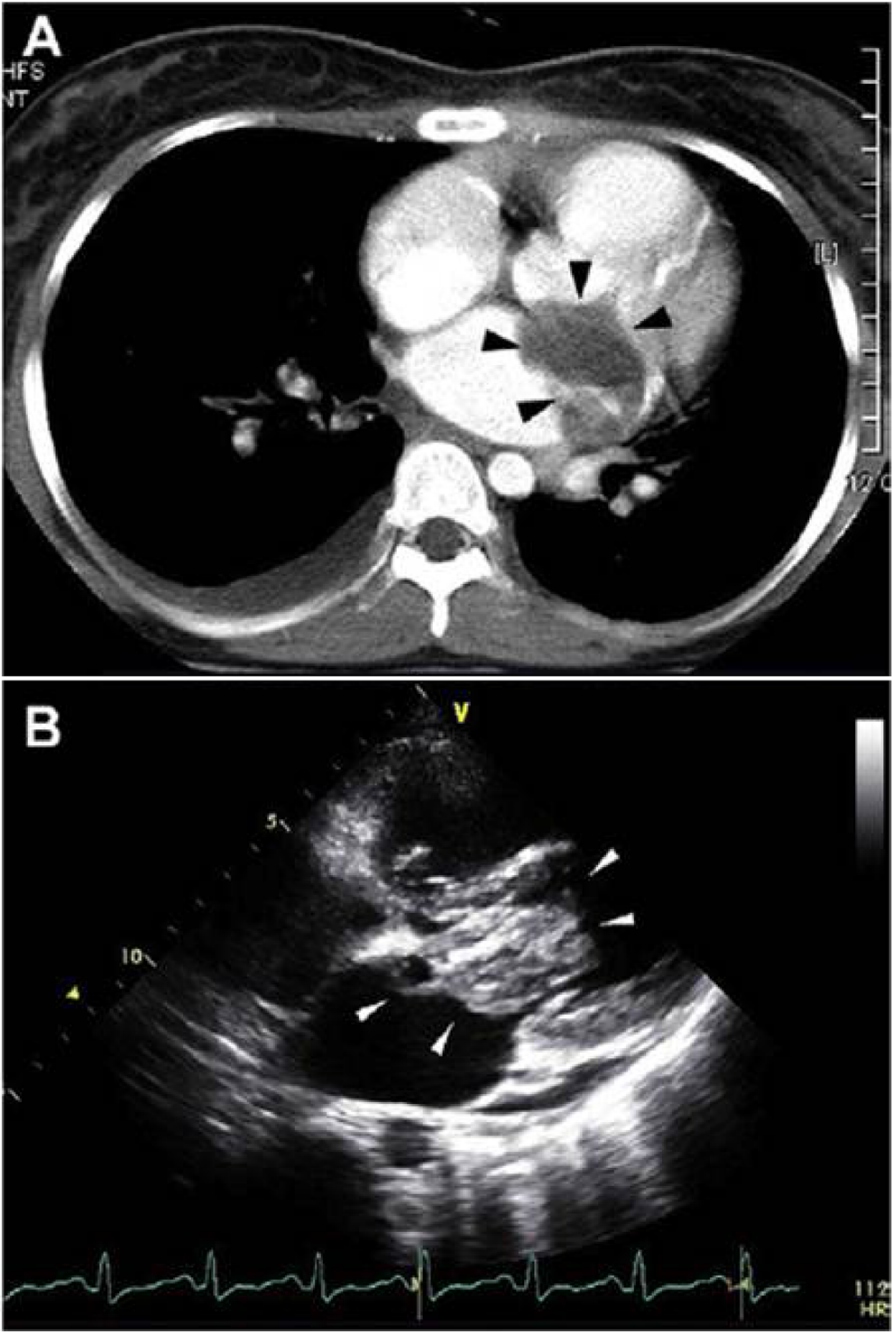
Rhabdomyosarcoma. **a**. Non-gated contrast CT demonstrating a large mass in the left atrium with heterogenous contrast enhancement (black arrows). **b**. Transthoracic echocardiogram demonstrates a large mass involving the mitral valve apparatus. Pathology was consistent with a rhabdomyosarcoma. *Reproduced with permission from [25]

Rhabdomyosarcomas appear as large, infiltrative masses that may surround a central area of necrosis on cardiac CT (Fig. 6). They may invade the epicardial fat, valves, or surrounding myocardium. The masses are intracavitary with irregular borders and low attenuation that may appear as thickening of the myocardium [10]. They may also appear as a hemorrhagic mass replacing the pericardium. Cardiac CT is useful for identifying any extracardiac extension of the tumor as well as distant metastasis, common to the lung parenchyma, thoracic lymph nodes, mediastinum, or spine [22].

## Non-Neoplastic Cardiac Masses

### Lipomatous Hypertrophy of the Interatrial Septum

**Fig. 7:**
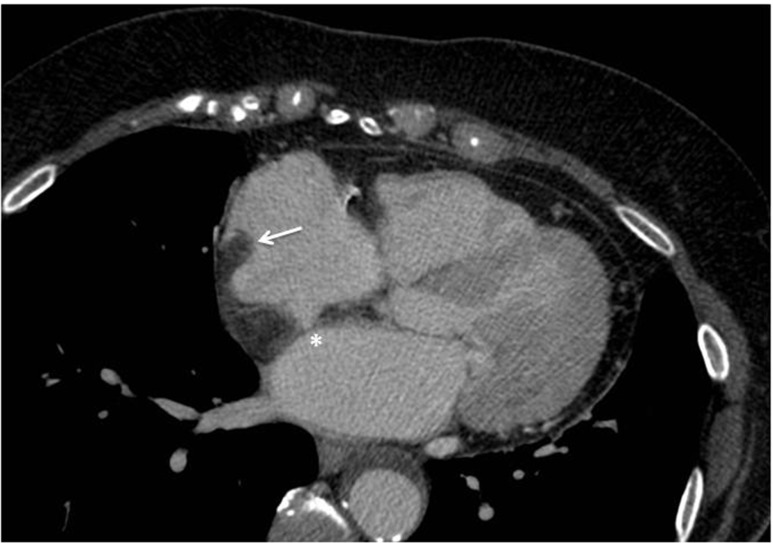
Lipomatous hypertrophy of the interatrial septum**.** Cardiac CT demonstrating a mass with fat-attenuation which spares the fossa ovalis (*) consistent with lipomatous hypertrophy of the interatrial septum. Note that the mass also extends to involve the posterior wall of the right atrium and crista terminalis (arrow), which may be seen in cases of more extensive lipomatous hypertrophy

Lipomatous hypertrophy of the interatrial septum (LHIAS) is an increasingly recognized, benign incidental finding with a prevalence of 1-8 % in the general population [24]. Incidence increases with age and body mass index. It occurs more frequently in women and those with a history of systemic steroid use [24]. Findings consistent with LHIAS on cardiac CT include a “dumbbell-shaped” mass involving the interatrial septum with sparing of the fossa ovalis, a homogenous appearance with sharp margins, fatty attenuation (density < -50 HU), and no or minimal contrast enhancement (Fig. 7). Lipomatous hypertrophy of the interatrial septum typically follows a benign course though associations with supraventricular arrhythmias, sudden cardiac death, and extensive impairment on venous return requiring surgical resection have been described [24, 26].

Although there is no definitive diagnostic criteria for the diagnosis of LHIAS, an interatrial septal thickness >20 mm is often used, while a septal thickness exceeding 30 mm may have a greater association with supraventricular arrhythmias [27]. It is important to note that areas of lipomatous hypertrophy contain brown fat and, therefore, may appear as FDG-avid (“hot”) on PET scans. Though rare, liposarcoma should be considered as a differential diagnosis, especially when intra-mass calcification or enhancement is present or in cases with rapid growth and involvement beyond the interatrial septum. Other causes of fat containing cardiac lesions include fatty metaplasia due to remote myocardial infarction, cardiac lipoma and arrhythmogenic right ventricular cardiomyopathy/dysplasia.

### Intracardiac Thrombus

Thrombus accounts for the most commonly encountered intracardiac mass [28]. It can occur in any of the cardiac chambers, though it most often involves the left-sided structures. Thrombus formation can be caused by hypercoagulable states, systolic dysfunction with wall motion abnormalities, atrial fibrillation, or artificial devices. It typically appears as a hypodense, low-attenuation filling defect in a contrast pool within a cardiac chamber and may be differentiated from primary and secondary tumors by knowledge of predisposing risk factors, attachment location, shape, and lack of mobility [29•].

Cardiac CT has very high sensitivity for excluding thrombus of the left atrial appendage but findings of low attenuation in the left atrial appendage (LAA) are not specific to thrombus as this often represents circulatory stasis, an incomplete mixing of contrast material and blood. This “pseudo” filling defect may mimic thrombus, especially in low-flow states [30•]. However, delayed imaging of the LAA may significantly improve the specificity to distinguish thrombus from circulatory stasis [31]. For example, a ratio of LAA attenuation to the ascending aorta > 0.75 on delayed cardiac CT images performed 30 seconds following first-pass contrast imaging can help differentiate thrombus from circulatory stasis with a high negative predictive value [32].

**Fig. 8:**
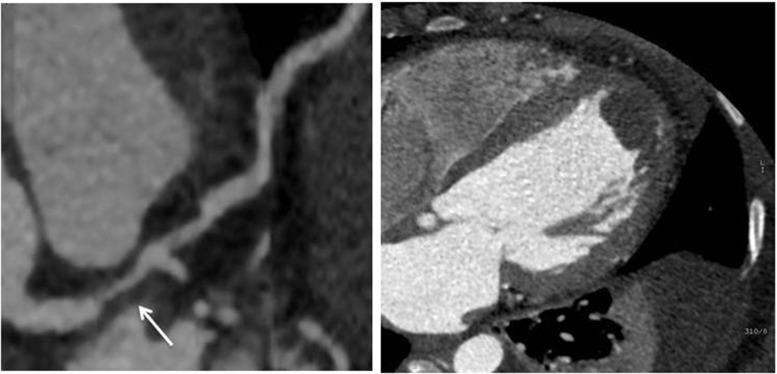
Left ventricular thrombus. Curved multiplanar image demonstrating a severe stenosis in the ostial portion of the left anterior descending artery (arrow) due to non-calcified plaque. **b**. Axial image from the same patient demonstrating a large hypodensity in the left ventricular apex consistent with thrombus

Left ventricular thrombi are often located in an area of myocardial hypokinesis, dyskinesia or aneurysm formation. They are frequently crescent-shaped filling defects with broad based attachments (Fig. 8). However, a pedunculated appearance has been observed and can mimic myxoma [33]. Chronic thrombi may develop spotty calcifications, though this feature has not been shown to significantly differentiate thrombus from myxoma. Thrombus within the left ventricle may be distinguished from myocardium by lower attenuation characteristics with a threshold of 65 HU providing a sensitivity and specificity of 94 % and 97 %, respectively [29•]. Right ventricular thrombus is a rare finding and usually associated with intravenous catheters, pulmonary embolism, or pulmonary embolism in transit. They have also been associated with arrhythmogenic right ventricular dysplasia, Bechet’s disease, metastatic disease, and trauma [28].

**Fig. 9:**
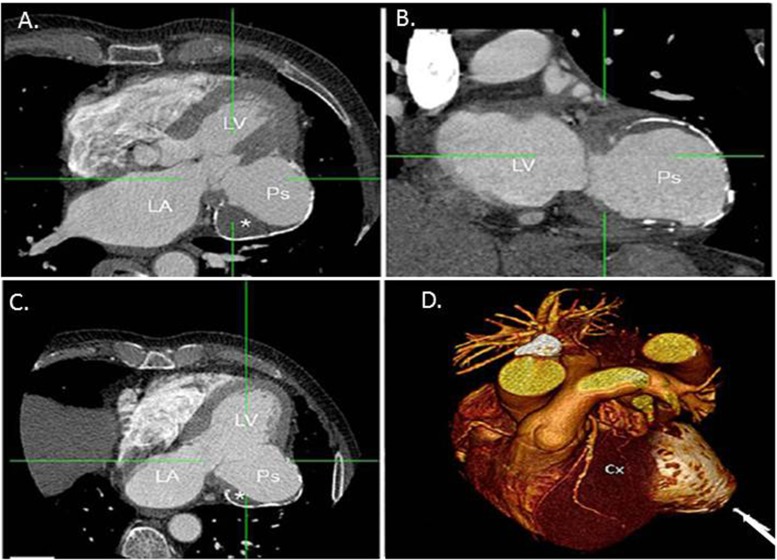
Left ventricular pseudoaneurysm. Contrast cardiac CT demonstrating a large pseudoaneurysm (Ps) of the left ventricular (LV) basal lateral wall as seen in oblique (**a**-**c**) and volume rendered (**d**) reformations. The patient had a history of a remote myocardial infarction that was complicated by cardiac tamponade due to left ventricular rupture. Note: thrombus (*) is visualized within the pseudoaneurysm and the rim of the pseudoaneurysm is calcified. LA, left atrium; Cx, circumflex coronary artery. *Reproduced with permission from [34]

In addition to intracardiac thrombus formation, the development of left ventricular pseudoaneurysm as a complication of a large myocardial infarction may present as a cardiac mass on echocardiography and can be easily distinguished using cardiac CT (Fig. 9).

### Pericardial Cyst

**Fig. 10:**
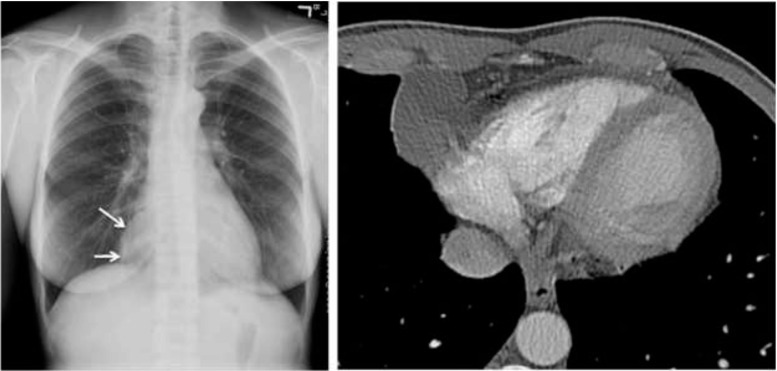
Pericardial cyst. (*left*) anterior chest X-ray suggestive of a mass at the right costophrenic angle. (*right*) contrast cardiac CT demonstrating that the mass correlates to a homogenous cyst in the right cardiophrenic angle most consistent with a benign pericardial cyst

Pericardial cysts are benign, congenital lesions that account for 7 % of all mediastinal tumors [35]. Over 75 % are located within the cardiophrenic spaces, the majority of which have a right-sided predominance. This anatomic location helps to distinguish pericardial cysts from other similar findings to include bronchogenic cysts, thymic cysts, and cardiac teratomas. Additionally, with pericardial cysts, there is no connection with the pericardial space, unlike that of pericardial diverticulae. On cardiac CT, pericardial cysts are thin-walled structures that are sharply demarcated, lack septae, and have a homogenous appearance (Fig. 10). They are non-enhancing lesions with intravenous contrast administration and have attenuation similar to water (-10 to 20 HU) [36].

### Valvular Vegetation

**Fig. 11:**
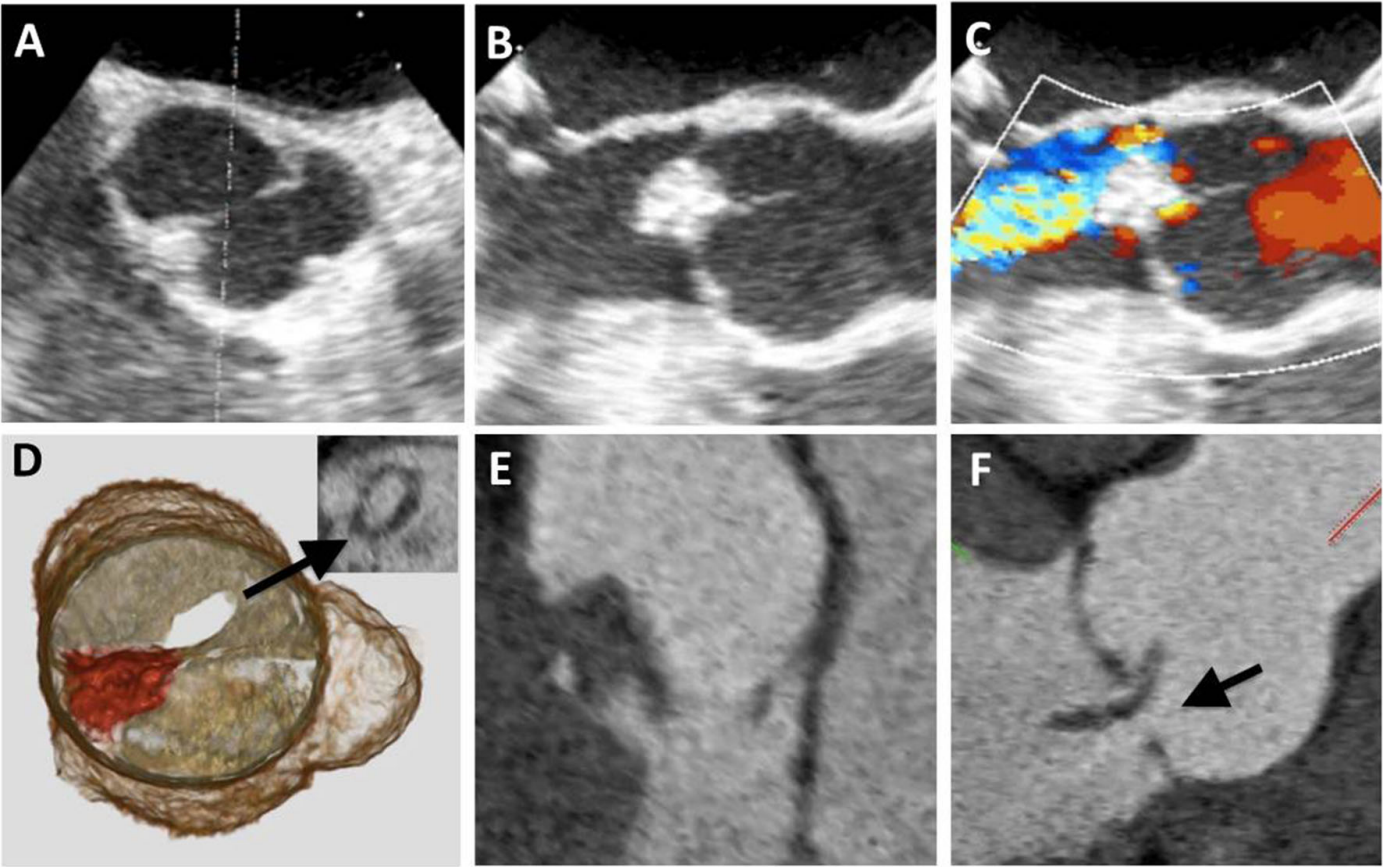
Valvular vegetation. Transesophageal echocardiogram (TEE), mid-esophageal short-axis (**a**) and long axis (**b**) views at peak diastole demonstrating a bicuspid aortic valve with fusion of the left and right coronary cusps and a 1.5 × 1.0 cm mobile echodensity attached to the right and non-coronary cusp. TEE long axis view with color Doppler demonstrates severe, eccentric aortic insufficiency (**c**). Cardiac CT 3D image reconstruction of a closed bicuspid aortic valve demonstrates the relationship of the mass (depicted in red) at the right and the non-coronary cusp with perforation of the non-coronary cusp (arrow) reproduced on 2D CCT short axis view (insert) with a regurgitant orifice area measuring 0.8 mm^2^ consistent with severe aortic regurgitation (**d**). Orthogonal 2D CCT 3-chamber views demonstrate a hypodense, irregular mobile mass (**e**) with associated flail leaflet and valve perforation (arrow) (**f**)

Cardiac CT has demonstrated to be highly accurate in identifying valvular vegetations, with a sensitivity of 97 % and specificity of 88 % as compared to transesophageal echocardiography [37]. On cardiac CT, vegetations appear as low-attenuation masses usually involving the valve leaflet free edge (Fig. 11). As a result, vegetations can lead to valvular destruction and/or dysfunction [38] and cardiac CT is particularly helpful in identifying perivalvular complications, such as perivalvular abscess formation, valve perforation, aortic pseudoaneurysms or extracardiac manifestations, such as septic emboli to the lung parenchyma.

### Crista Terminalis

**Fig. 12:**
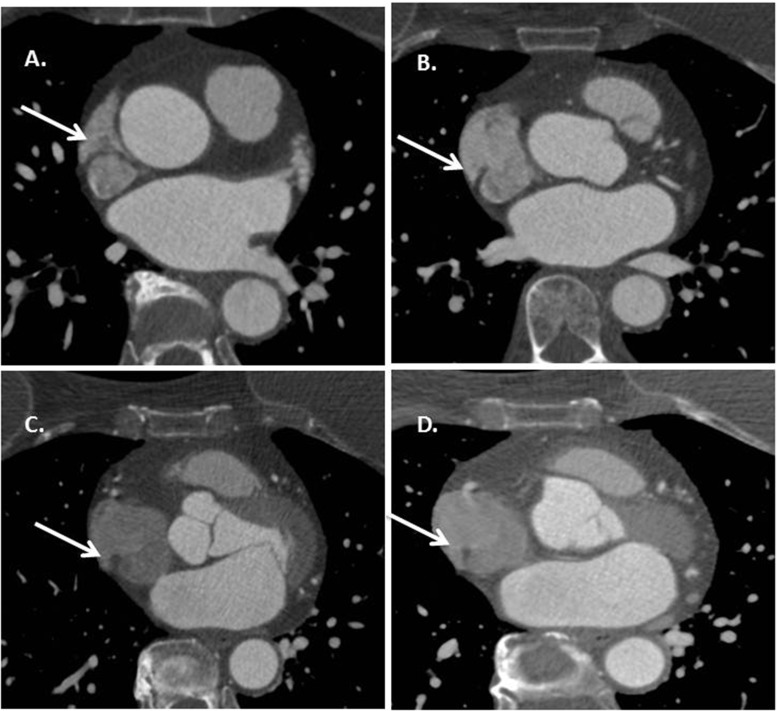
Crista terminalis. Serial axial images (**a**-**d**) of a coronary CT angiography study demonstrating the normal appearance of the crista terminalis (arrow) extending in the cranio-caudal region in the posterior wall of the right atrium

The crista terminalis is a common mimicker of cardiac tumors when seen on cardiac CT and can be mistaken for a right atrial mass or thrombus. The crista terminalis is a smooth-surfaced, linear fibromuscular structure, with a vertical orientation and crescent shape running along the posterior wall of the right atrium from the superior vena cava to the inferior vena cava (Fig. 12). It separates the smooth sinus venosus portion of the right atrium from the trabeculated right atrial tissue and right atrial appendage. Regression is inconsistent among individuals resulting in a significant variation in the degree of fibromuscular extension. It is most notable when triphasic contrast injection protocols are used for cardiac CT. It can be distinguished on cardiac CT as a focal, low-density attenuation similar to fat.

## Conclusion

In summary, cardiac CT can provide useful anatomic and functional information as an adjunct to echocardiography and MR in the evaluation of cardiac masses. With high spatial and contrast resolution, fast acquisition times, and the capability to identify calcification and fat, cardiac CT can serve as an ideal alternative to MR imaging, especially in patients with contraindications. Furthermore, cardiac CT may have specific advantages in defining the cardiovascular extent of the mass and excluding coronary artery disease prior to surgical intervention. With the continued, widespread utilization of cardiac CT, it is important to accurately distinguish cardiac masses in order to provide optimal medical management.
